# Induction of senescence in primary glioblastoma cells by serum and TGFβ

**DOI:** 10.1038/s41598-017-02380-1

**Published:** 2017-05-19

**Authors:** Ritesh Kumar, Alexander Gont, Theodore J. Perkins, Jennifer E. L. Hanson, Ian A. J. Lorimer

**Affiliations:** 1 Cancer Therapeutics Program, Ottawa Hospital Research Institute, 501 Smyth Road, Ottawa, K1H 8L6 Canada; 2 Department of Biochemistry, Microbiology and Immunology, University of Ottawa, Ottawa, Ontario Canada; 3 Department of Surgery, University of Ottawa, Ottawa, Ontario Canada; 4 Regenerative Medicine Program, Ottawa Hospital Research Institute, 501 Smyth Road, Ottawa, K1H 8L6 Canada; 5 Department of Medicine, University of Ottawa, Ottawa, Ontario Canada

## Abstract

Glioblastoma is the most common type of adult brain tumour and has a median survival after diagnosis of a little more than a year. Glioblastomas have a high frequency of mutations in the TERT promoter and CDKN2A locus that are expected to render them resistant to both replicative and oncogene-induced senescence. However, exposure of PriGO8A primary glioblastoma cells to media with 10% serum induced a senescence-like phenotype characterized by increased senescence-associated β galactosidase activity, PML bodies and p21 and morphological changes typical of senescence. Microarray expression analysis showed that 24 h serum exposure increased the expression of genes associated with the TGFβ pathway. Treatment of PriGO8A cells with TGFβ was sufficient to induce senescence in these cells. The response of PriGO8A cells to serum was dependent on basal expression of the TGFβ activator protein thrombospondin. Primary glioblastoma cells from three additional patients showed a variable ability to undergo senescence in response to serum. However all were able to undergo senescence in response to TGFβ, although for cells from one patient this required concomitant inhibition of Ras pathway signalling. Primary glioblastoma cells therefore retain a functional senescence program that is inducible by acute activation of the TGFβ signalling pathway.

## Introduction

Glioblastoma is an aggressive form of brain cancer. A characteristic feature of glioblastoma is its heterogeneity. This was originally observed in its histology, giving rise to the term glioblastoma multiforme. More recently genetic studies have created a detailed picture of extensive heterogeneity at the molecular level. Analysis of microarray expression data has led to the subdivision of glioblastoma into four or five different molecular subtypes, designated G-CIMP/proneural, neural, classical and mesenchymal^1^. These tend to be associated with different mutations with, for example, *EGFR* being frequently amplified and mutated in the classical subtype and *NF1* being frequently mutated in the mesenchymal subtype. Single cell analysis has shown that most or all glioblastomas contain more than one subtype, with the proneural being present to some degree in all patients tested^2^. This and additional data^3^ suggest that several other glioblastoma subtypes evolve from the proneural subtype through the acquisition of additional mutations.

Neither radiation nor current chemotherapy is curative in glioblastoma. It has been proposed that this is due to a high capacity for DNA repair in a subset of glioblastoma cells with stem cell-like features^4, 5^. Radiation and chemotherapy can lead to various outcomes in cancer cells, including cell death or senescence^6, 7^. Senescence is a state of irreversible growth arrest in cells^8, 9^. Senescent cells show morphologic changes that include flattening, enlargement of the cytoplasm and increased cytoplasmic granularity^6, 10, 11^. They also show characteristic biochemical changes including an increase in senescence-associated β-galactosidase (SAβgal) activity^12^ and an increase in PML bodies in the nucleus^13^. Senescence can be divided into two types. Replicative senescence is triggered by the loss of telomeric repeats from the ends of chromosomes after multiple cell divisions. Over 80% of glioblastoma cells have telomerase promoter mutations that allow them to bypass replicative senescence^14, 15^. Premature senescence is a second type of senescence that occurs in the absence of telomere erosion^6^. This can be induced by a variety of cell stresses including oxidative stress, replicative stress, radiation and some chemotherapeutic agents. Premature senescence can also be induced by oncogenes – in this context it is thought to be an important endogenous mechanism for cancer prevention^16^. Premature senescence has previously been studied in glioblastoma cell lines^17–20^ and primary cultures isolated in serum^21^. This has shown that senescence can occur by both p53-dependent and -independent mechanisms^20^. Much current glioblastoma research is focused on the use of primary glioblastoma cells isolated under serum-free conditions. Unlike glioblastoma cell lines, these cells accurately model glioblastoma behaviour when grown orthotopically in immunocompromised mice. They also show many neural stem cell-like characteristics, including the expression of nestin and sox2, and the ability to undergo differentiation along multiple lineages. Serum exposure is known to reverse many of these stem cell-like properties. The Fine lab has published a detailed study on the effects of long-term culture in the presence of serum on glioblastoma cells isolated from patients^22^. These included: altered morphology; altered growth kinetics; aberrant differentiation; transient loss of telomerase activity, loss of tumorigenic potential, altered gene expression profiles and genomic rearrangements. While that study described in detail the long-term consequences of serum exposure, the signalling pathways that drive this response to serum were not assessed. As well, the issue of why primary glioblastoma cells behave this way, while standard glioblastoma cell lines are readily grown in the presence of serum, was not addressed. Here we have studied the short term responses of primary glioblastoma cells to serum, showing that serum induces a thrombospondin 1-dependent activation of TGFβ pathway signalling in these cells. Acute activation of the TGFβ pathway is able to induce senescence of primary glioblastoma cells from multiple patients.

## Results

### Serum induces abnormal differentiation

**Figure 1:**
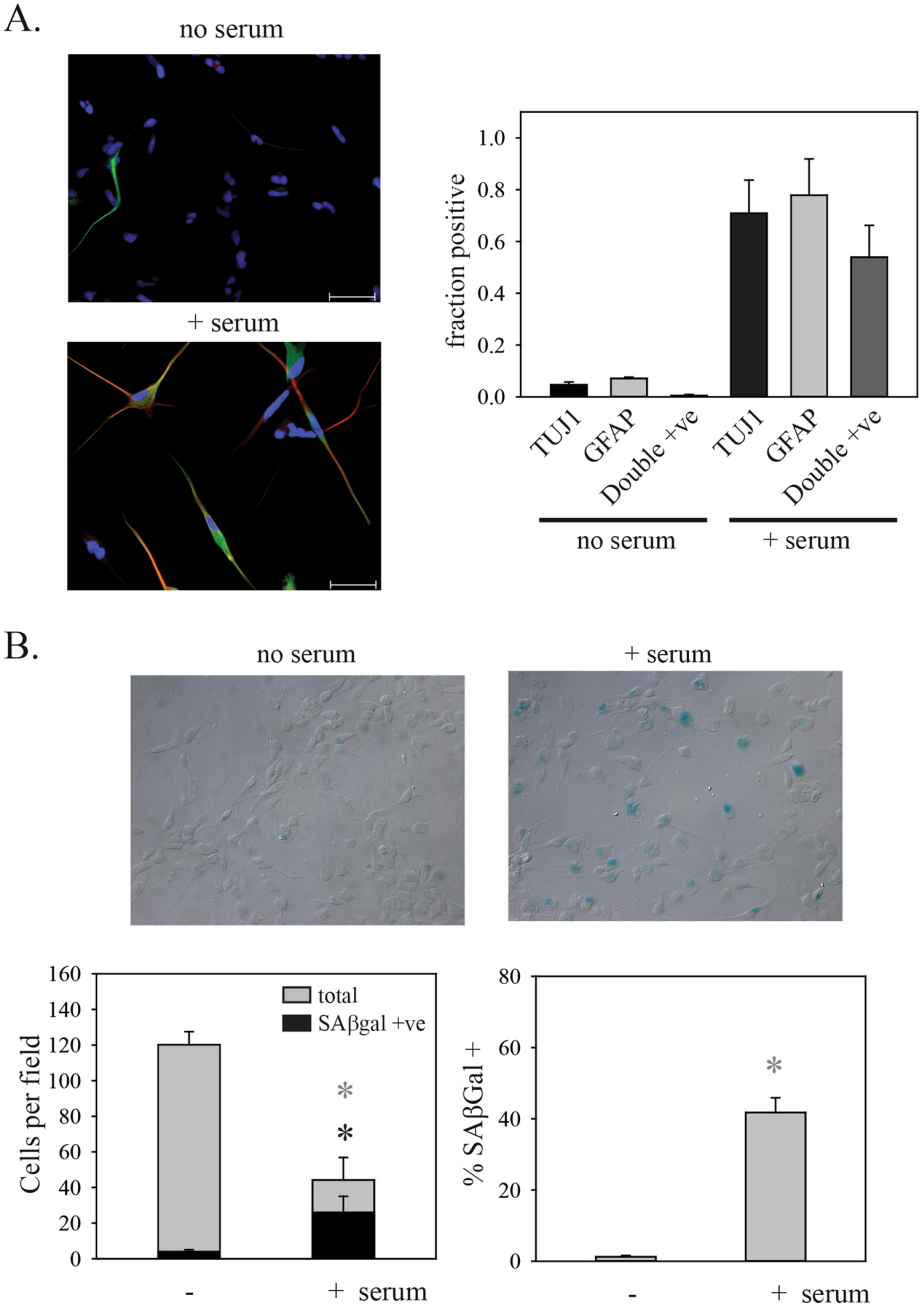
Serum induces aberrant differentiation and senescence. (**A**) PriGO8A cells were untreated or treated with 5% serum for seven days. Cells were then fixed and double immunofluorescence for GFAP (green) and TUJ1 (red) was performed. Nuclei were stained with DAPI (blue). Left panels show representative examples of immunofluorescence. The right panel show quantitation of the numbers of positive cells. (**B**) PriGO8A cells were untreated or treated with 10% serum for seven days. Cells were then fixed and stained for SAβgal activity. The upper panels show representative images of cells stained for SAβgal after treatment without or with serum. Images were taken using differential interference contrast microscopy. The right graph shows total cells (light grey bars) and SAβgal-positive cells per field. The left graph shows the same data plotted to show the change in the percentage of SAβgal-positive cells. For (**A** and **B**), data are from analysis of five random fields per condition. Error bars show the standard error. In (**B**), statistical significance was determined using the Mann-Whitney Rank Sum test. *Indicates p < 0.05.

PriGO8A cells are a primary glioblastoma cell line isolated in serum-free media as described previously^23^. They show stem cell-like features in that they express nestin and sox2 and are able to grow as neurospheres when cultured in the absence of a laminin substrate. They do not express p16 or PTEN. Exposure to serum induces an apparent differentiation of these cells, as the number of cells expressing markers of differentiation along the neuronal (TUJ1) and astrocytic (GFAP) lineages. However this differentiation is abnormal in that about 50% of cells are double positive for TUJ1 and GFAP (Fig. 1A).

### Serum induces senescence

**Figure 2:**
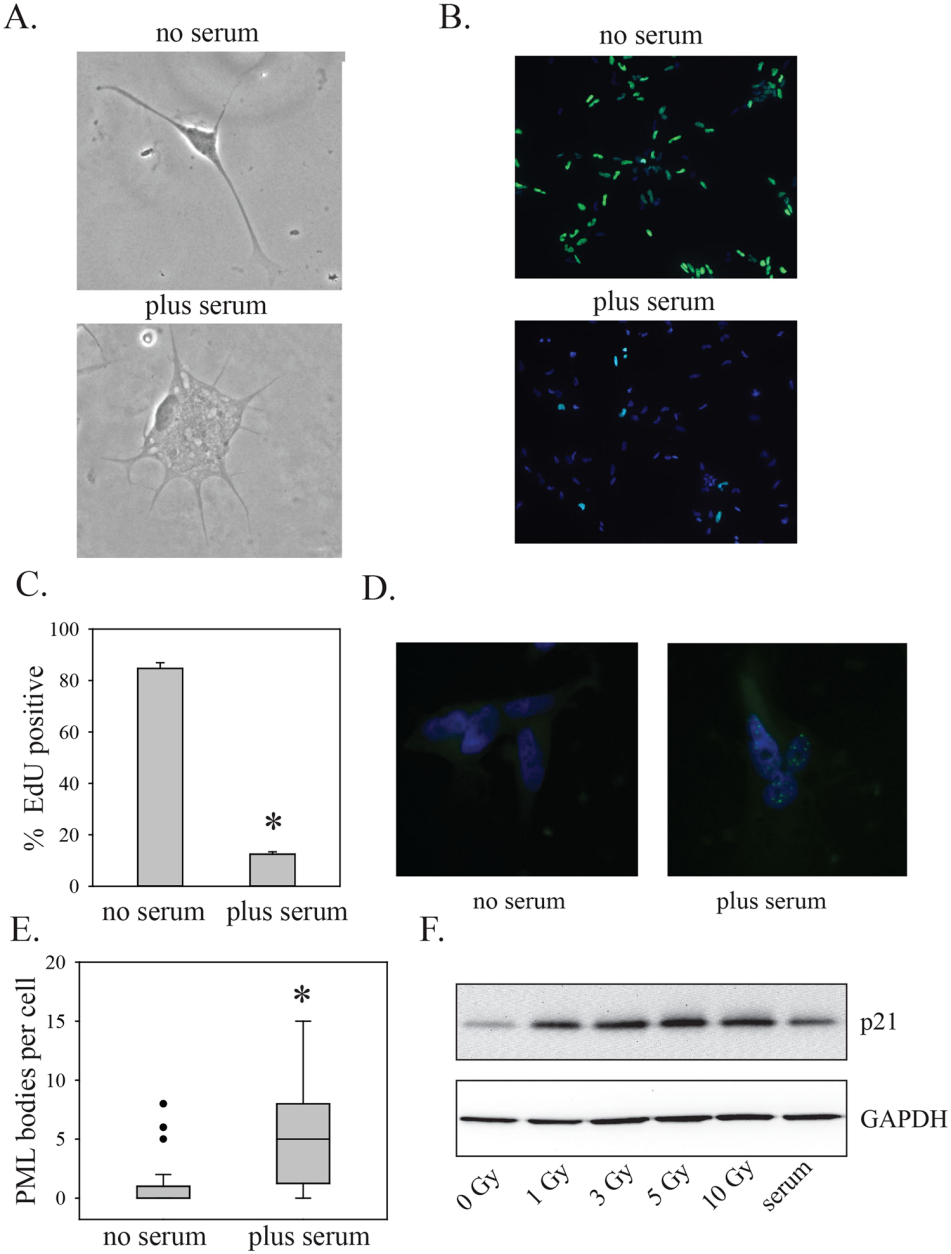
Characterization of senescence induction by serum. (**A**) PriGO8A cells were treated without or with serum for seven days. Cells were then fixed and stained for SAβgal activity. Images shown are representative phase contrast images of SAβgal-cells (photographed in black and white). (**B**) PriGO8A cells were treated as in (**A**). On day 7, they were labelled for 24 h with EdU. Cells with EdU incorporation were detected as described in Materials and Methods and nuclei were stained with DAPI. (**C**) Quantitation of EdU incorporation. Data are from five randomly chosen fields. (**D**) PriGO8A cells were treated as in (**A**), fixed and analyzed for PML bodies by immunofluorescence. Representative images are shown with PML in green and DAPI staining in blue. (**E**) Quantitation of PML immunofluorescence. Data are from 100 cells per condition. Whiskers show 10^th^ and 90^th^ percentiles. Statistical significance was determined using the Mann-Whitney Rank Sum test. *Indicates p < 0.05. (**F**) PriGO8A cells were treated with the indicated doses of radiation or 10% serum. Two days later, total cell extracts were analyzed by Western blotting for levels of p21. GAPDH was used as a loading control.

**Figure 3:**
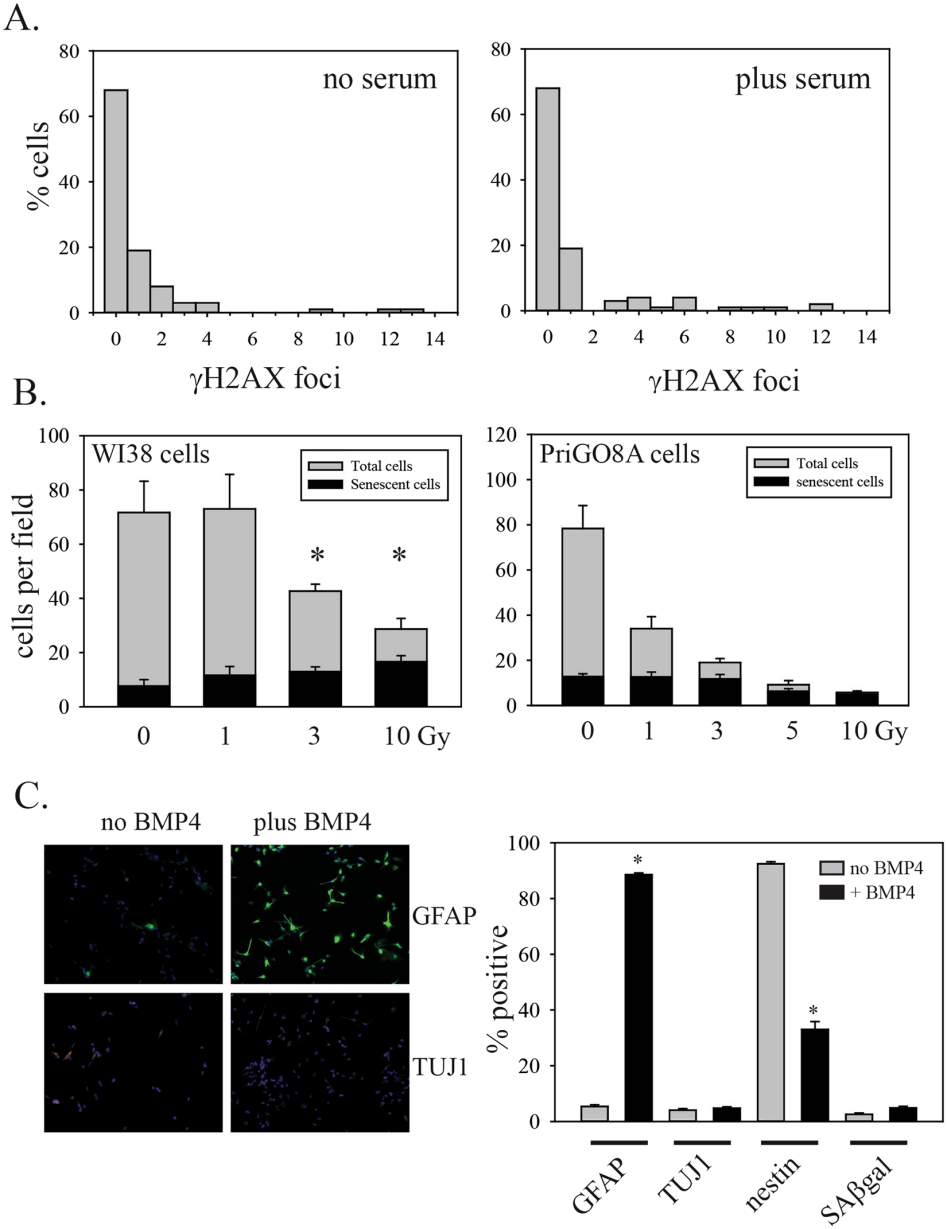
DNA damage and senescence and BMP4-induced differentiation. (**A**) PriGO8A cells were treated without or with serum for seven days. Cells were then fixed and analyzed for γH2AX foci by immunofluorescence. Data are from approximately 100 cells per condition. The percent of cells with different numbers of foci from 0 to 14 foci per nucleus are plotted. (**B**) WI38 normal human fibroblasts and PriGO8A cells were exposed to the indicated doses of radiation. Seven days later, cells were fixed and stained for SAβgal and total and senescent cells were counted in randomly chosen fields. Statistical significance was determined using two-tailed t tests. *Indicates p < 0.05 for a change in senescent cell numbers relative to 0 Gy radiation. Representative images of PriGO8A and WI38 γH2AX immunofluorescence are shown in Supplementary Figure S1. (**C**) PriGO8A cells were treated without or with 100 ng/ml BMP4. Seven days later cells were fixed and assayed for GFAP, TUJ1 and nestin expression by immunofluorescence. SAβgal assays were performed as described in Materials and Methods. Representative images of GFAP and TUJ1 immunofluorescence are shown. The bar graph shows quantitation of GFAP, TUJ1, nestin and SAβgal positive cells. Data are from five randomly selected fields per condition. Statistical significance was determined using two-tailed t tests. *Indicates p < 0.05.

To determine if serum induced senescence features, we first assayed for SAβgal, one of the most widely used markers of senescence^12^. PriGO8A cells showed an increase in the percentage of cells that were SAβgal positive after exposure to serum for seven days (Fig. 1B). Serum-treated PriGO8A cells also showed the flattened morphology and granular cytoplasm that is characteristic of adherent senescent cells (Fig. 2A) and a decrease in EdU incorporation, indicative of growth arrest (Fig. 2B and C). Serum-treated PriGO8A cells also showed an increase in nuclear PML bodies (Fig. 2D and E), a feature that is associated with both replicative and oncogene-induced senescence in human fibroblasts^13^. Serum also caused a rise in p21, a negative regulator of the cell cycle that is often present at high levels in senescent cells, as well as quiescent cells^6^ (Fig. 2F). These features were seen in the absence of a detectable increase in γH2AX foci (Fig. 3A and Supplementary Figure S1). Radiation treatment rapidly induced cell death in PriGO8A cells (Figure S1B) but did not increase numbers of senescent PriGO8A cells above levels seen in control conditions (Fig. 3B). This is similar to findings reported previously for p53-deficient glioblastoma cell lines, where DNA damage with a topoisomerase I inhibitor induced massive cell death rather than senescence^21^.

### BMP4 induces astrocytic differentiation without markers of senescence

In agreement with previously described findings with primary glioblastoma cells, treatment with BMP4 induced differentiation of PriGO8A cells along the astrocytic lineage, with no significant differentiation along the neuronal lineage or apparent aberrant differentiation^24^ (Fig. 3C). This was accompanied by a decrease in nestin positivity (Fig. 3C). BMP4 did not cause an increase in SAβgal positive cells (Fig. 3C).

### Microarray gene expression analysis of primary glioblastoma cell senescence

**Figure 4:**
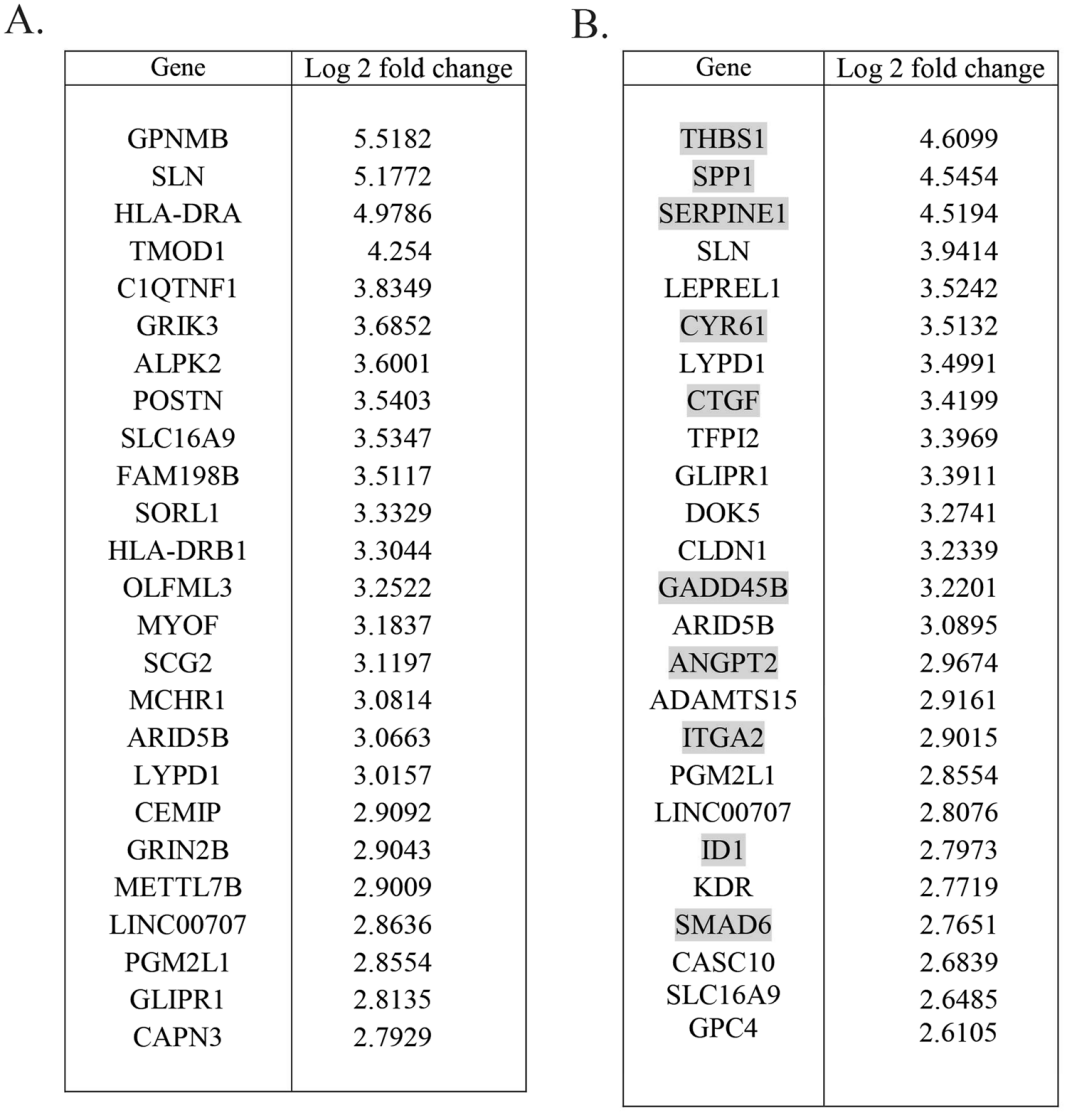
Long term and short term effects of serum on gene expression. PriGO8A cells were treated without or with 10% serum for either seven days (**A**) or 24 h (**B**). RNA was then isolated and microarray expression analysis performed. The 25 genes showing the largest increases in expression with serum are shown. In (**B**), genes that have previously been shown to be regulated by TGFβ are highlighted in grey.

Microarray expression analysis was used to compare gene expression profiles between untreated PriGO8A cells and PriGO8A cells exposed to serum for 24 h or seven days. For the day seven data, 406 genes showed changes in gene expression greater than two-fold relative to the untreated control. The top 25 genes are shown in Fig. 4A. Pathway analysis with Enrichr^25^ and KEGG^26^, or Wikipathways 2015^27^ identified a strong extracellular matrix/focal adhesion/integrin-mediated cell adhesion pathway component in the senescent phenotype. There was no obvious senescence-associated secretory phenotype, with the mRNA expression of cytokines such as IL6 and IL8 being unchanged^28^.

### Microarray gene expression analysis of serum response

Analysis of expression changes after 24 h of serum exposure showed that the expression of 261 genes was increased by two-fold or more. The top 25 genes are shown in Fig. 4B. Pathway analysis with multiple bioinformatics analysis methods (DAVID Bioinformatics Resources 6.7^29, 30^ and KEGG pathway analysis^26^, Enrichr^25^ with Wikipathways 2015^27^ or Biocarta 2015 indicated that TGFβ pathway activation was a prominent signalling pathway activated with 24 h exposure to serum. Transcription factor analysis using Enrichr and ChEA^31^ identified SMAD2, SMAD3 and SMAD4 as candidate transcription factors mediating the changes in these 49 genes, consistent with TGFβ pathway activation and transcriptional regulation by the canonical TGFβ pathway. Previously, Nogueira *et al*. reported activation of the NFκB pathway in primary glioblastoma cells induced to differentiate with serum^32^. This was not evident in the analysis performed here.

### Serum activates TGFβ pathway signaling

**Figure 5:**
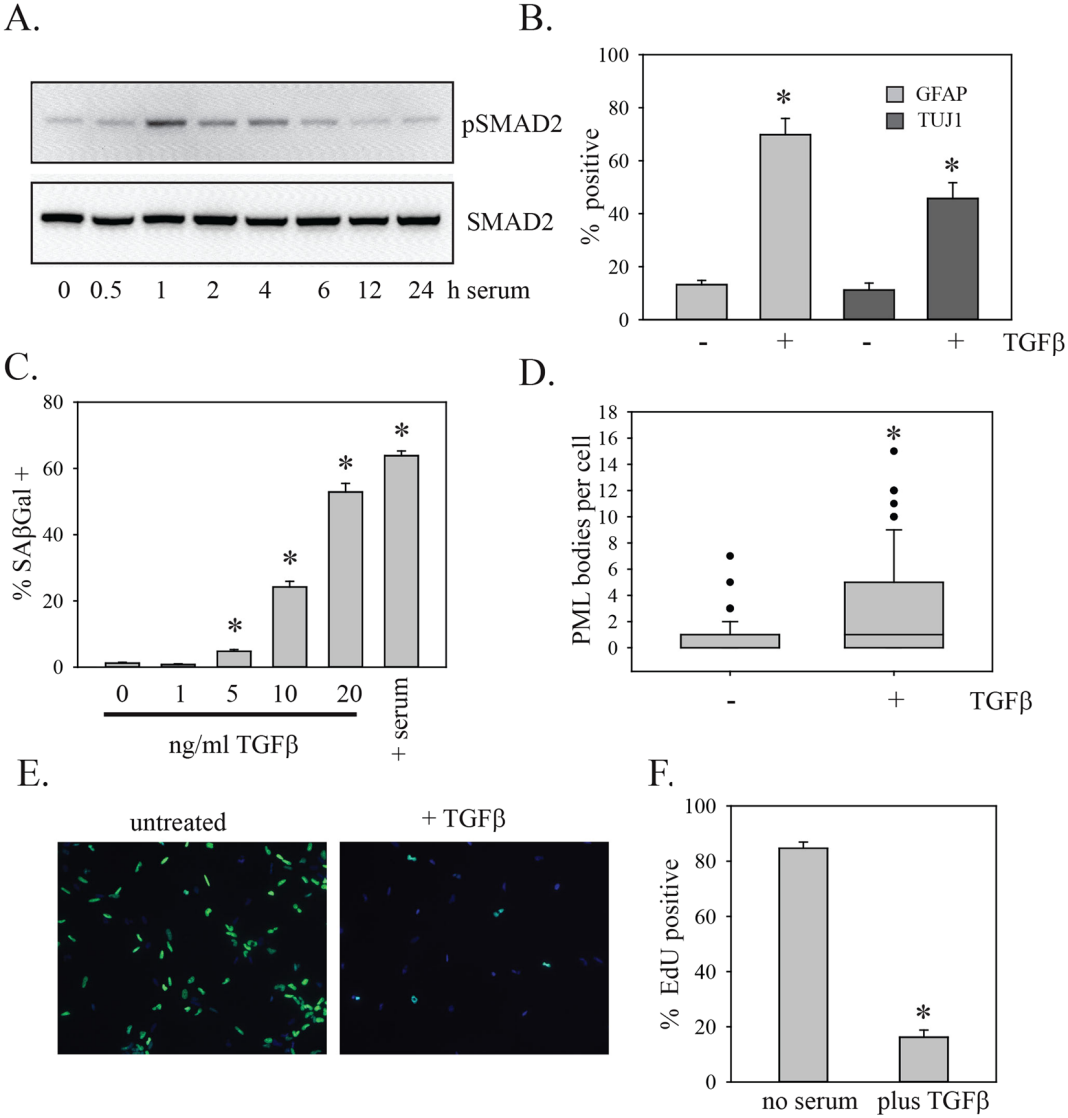
TGFβ effects on differentiation and senescence. (**A**) PriGO8A cells were treated with media without or with 10% serum for the indicated period of time. Total cell lysates were then assayed for phosphorylated and total SMAD2 by Western blotting. (**B**) PriGO8A cells were treated with 20 ng/ml TGFβ. Seven days later they were assayed for GFAP and TUJ1 expression as in Fig. 1. (**C**) PriGO8A cells were treated with the indicated concentrations of TGFβ. Seven days later they were assayed for SAβgal activity as in Fig. 1. (**D**) PriGO8A cells were treated with 20 ng/ml TGFβ. Seven days later they were fixed and analysed for PML bodies by immunofluorescence as in Fig. 2. (**E**) PriGO8A cells were treated as in (**B**) On day 6, they were labelled for 24 h with EdU. Cells with EdU incorporation were detected as described in Materials and Methods and nuclei were stained with DAPI. (**F**) Quantitation of EdU incorporation. Data are from five randomly chosen fields. For (**B** and **C**), statistical significance was determined with two-tailed t-tests. For (**D** and **F**), statistical significance was determined using the Mann-Whitney Rank Sum test. *Indicates p < 0.05 in comparison with the no TGFβ control data.

To confirm activation of the TGFβ pathway by serum, cells were treated with serum for varying periods of time and then assayed for phosphorylation of SMAD2. Increased SMAD2 phosphorylation was evident after 1 h of exposure to serum (Fig. 5A); these kinetics are similar to what is seen in cells exposed to TGFβ^33^. Treatment of PriGO8A cells with TGFβ also reproduced the effects of serum on differentiation and senescence markers (Fig. 5B–F).

### Thrombospondin 1 is required for serum effects on primary glioblastoma cells

**Figure 6:**
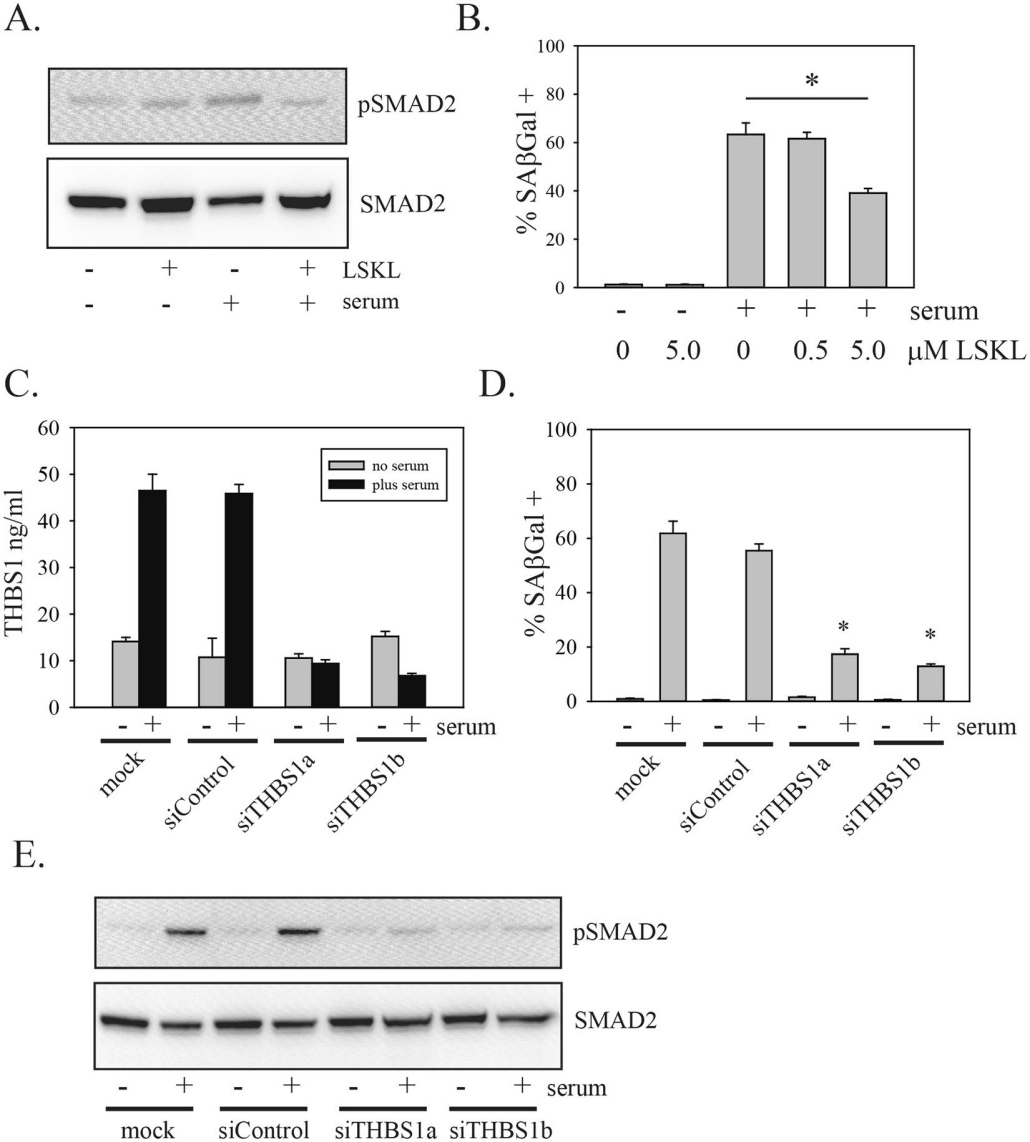
Thrombospondin is required for activation of the TGFβ pathway by serum. (**A**) PriGO8A cells were pretreated for 1 h without or with the thrombospondin inhibitor peptide LSKL as indicated. Cells were then treated with media without or with 10% serum for one hour. Total cell lysates were analyzed for phosphorylated and total SMAD2 levels by Western blotting. Representative results from one of three independent experiments are shown. (**B**) PriGO8A cells were treated with or without serum plus the indicated concentrations of LSKL peptide. Media with or without peptide was replaced every two days and SAβgal activity was determined after seven days. (**C**) PriGO8A cells were mock transfected, or transfected with control siRNA or two different siRNA targeting thrombospondin 1. 48 h after transfection, media was removed and replaced with media either without or with serum. Conditioned media samples were collected after 24 h and assayed for thrombospondin levels by ELISA. **(D)** PriGO8A cells were transfected as in (**C**) Two days later cells were treated without or with serum as indicated. SAβgal assays were performed 7 days after the initiation of serum treatment. (**E**) PriGO8A cells were transfected as in (**C**) 48 h later media was removed and replaced with fresh media without or with serum. One hour later total cell lysates were collected and analyzed for phosphorylated and total SMAD2 by Western blot.

The most highly induced mRNA in response to 24 h serum treatment was the mRNA encoding thrombospondin 1 (Fig. 4B). Previous studies have also identified thrombospondin 1 as a TGFβ-regulated gene^34^. In addition to being regulated by TGFβ at the mRNA level, thrombospondin is also well-known as an activator of latent TGFβ^35^. As PriGO8A cells express detectable amounts of thrombospondin basally, this provides a potential mechanism for the initial activation of latent TGFβ in serum^36, 37^, along with a positive feedback mechanism for further activation of latent TGFβ. Latent TGFβ is mature TGFβ complexed with a latency associated peptide. This complex is unable to bind receptor until activated by either protease activity or binding of the latency-associated peptide to another protein. Pretreatment of PriGO8A cells with the thrombospondin inhibitor peptide LSKL effectively blocked the activation of SMAD2 by serum (Fig. 6A). LSKL also partially inhibited the effects of serum on senescence induction (Fig. 6B). The partial inhibition is likely due to peptide instability. Knockdown of thrombospondin 1 using RNA interference (Fig. 6C) also prevented activation of the TGFβ pathway and senescence induction by serum in these cells (Fig. 6D and E).

### Response of primary glioblastoma cells from additional patients

**Figure 7:**
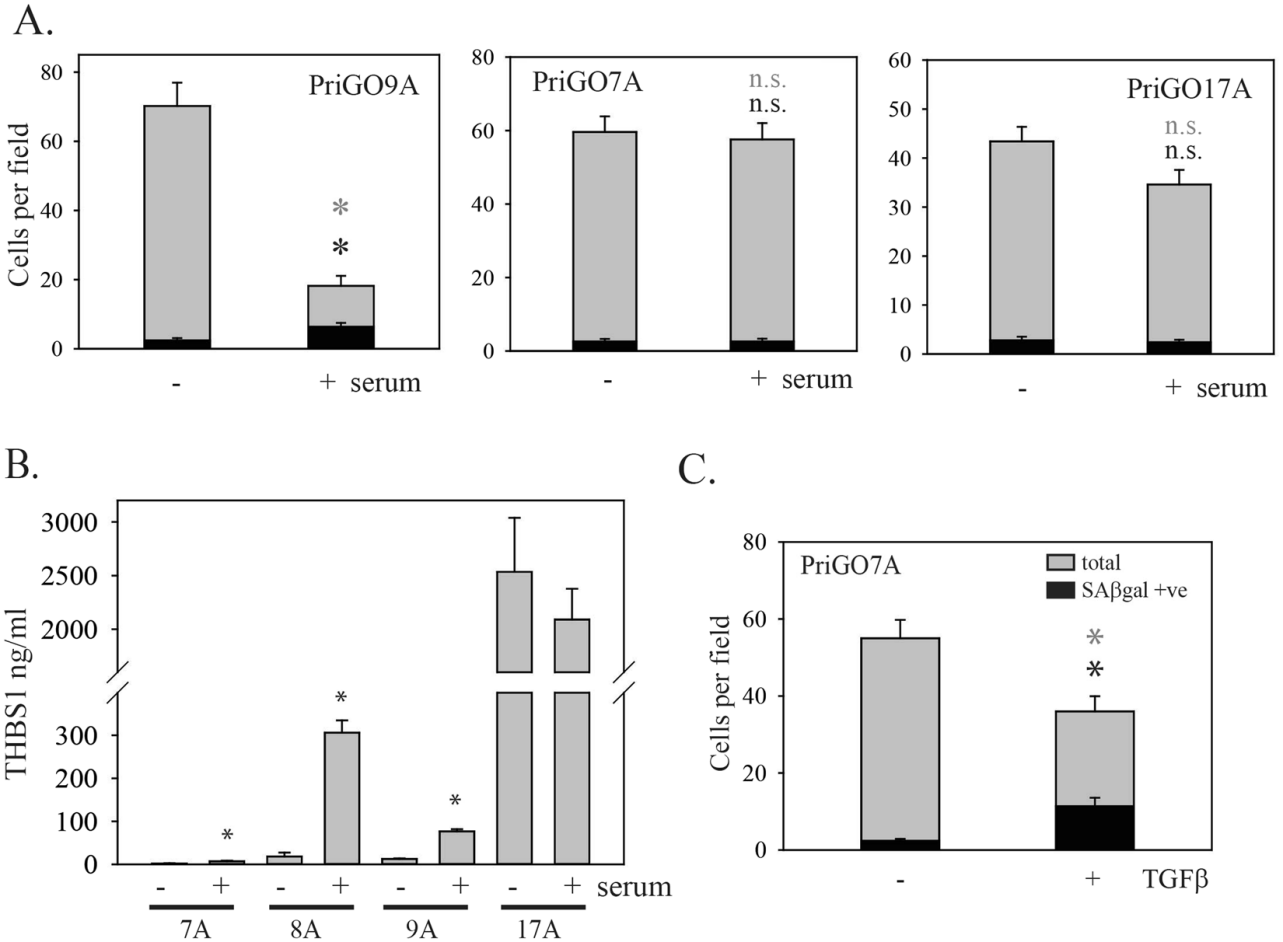
Heterogeneity of responses to serum in cells from different glioblastoma patients. (**A**) PriGO9A, PriGO7A and PriGO17A cells were treated with 10% serum and assayed for SAβgal activity as described in Fig. 1. Statistical significance was determined using two-tailed t tests. (**B**) PriGO cells from different patients were treated without or with serum for 24 h. Conditioned media was then collected and analyzed for levels of thrombospondin by ELISA. Data shown are mean ± SD for triplicate analyses for each condition and are corrected for background of either media alone or media plus serum, as well as corrected for cell number at the time of collection of conditioned media. Statistical significance was determined using the Mann-Whitney Rank Sum test. (**C**) PriGO7A cells were treated without or with 20 ng/ml TGFβ. Seven days later cells were fixed and stained for SAβgal activity. Data are from analysis of five random fields per condition. Error bars show the standard error. Statistical significance was determined using two-tailed t-tests. For (**A–C**), *indicates p < 0.05 and n.s. indicates p > 0.05 compared to no TGFβ control.

To assess the generalizability of the findings on PriGO8A cells, we determined whether serum was able to induce senescence in glioblastoma cells isolated from three additional patients. PriGO9A cells also showed senescence induction in response to serum (Fig. 7A and Supplementary Figure S1). However cells from two other patients did not (Fig. 7A and Supplementary Figure S1). PriGO7As expressed very little thrombospondin basally (Fig. 7B), suggesting that the lack of senescence induction might be due to an inability to activate latent TGFβ. Consistent with this, treatment of these cells with active TGFβ was able to induce senescence (Fig. 7C).

**Figure 8:**
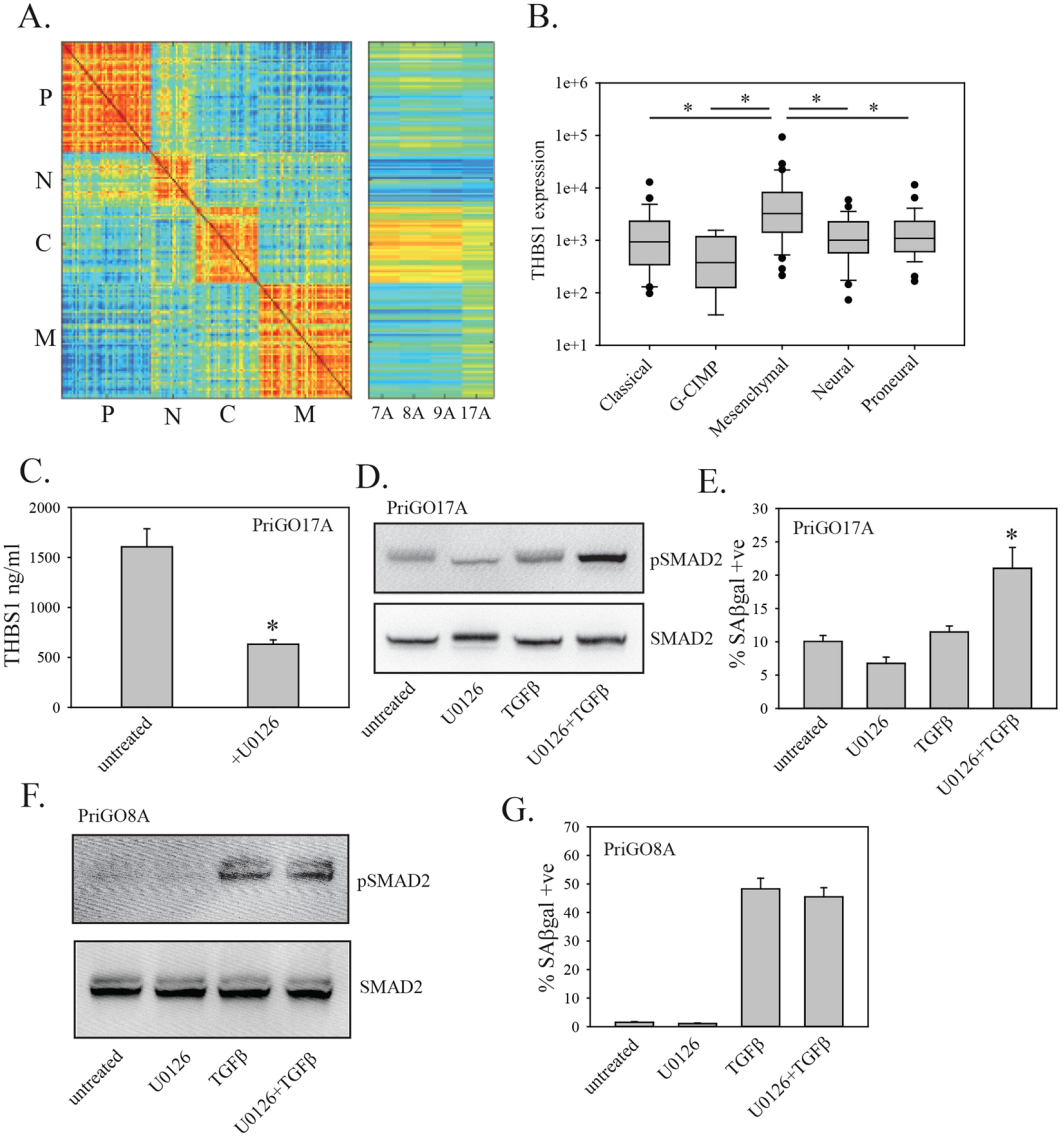
Molecular subtype of PriGO17A cells and effects of Ras pathway inhibition. (**A**) Glioblastoma cell lines were classified into molecular subtypes as described in Materials and Methods. (**B**) TCGA database analysis of thrombospondin 1 mRNA expression was performed using cBioportal and the TCGA glioblastoma 2013 database. Thrombospondin 1 mRNA expression data are plotted with respect to molecular subtype. Statistical significance was determined using the All Pairwise Multiple Comparison Procedures (Dunn’s Method). *Indicates p < 0.05. (**C**) PriGO17A cells were treated without or with 50 μM U0126 for 24 h. One day after plating, media was changed and inhibitor was added. Conditioned media was collected 24 h after adding the inhibitor and was assayed for levels of thrombospondin 1 by ELISA. (**D**) PriGO17A cells were pretreated for 24 h with 50 μM U0126 as indicated. Cells were then treated with 20 ng/ml TGFβ and total cell lysates were collected 1 h later. Levels of phosphorylated and total SMAD2 were analyzed by Western blotting. (**E**) PriGO17A cells were treated with U0126 and TGFβ as in (**D**). Seven days later, cells were fixed and assayed for SAβgal activity. (**F**) PriGO8A cells were treated and analyzed for SMAD2 phosphorylation as in (**D**). (**G**) PriGO8A cells were treated and analyzed for SAβgal activity as in (**E**) Error bars show the standard error. Statistical significance was determined using All Pairwise Multiple Comparison Procedures (Holm-Sidak method). *indicates a p ≤ 0.05 compared to all other groups.

In contrast to PriGO7A, PriGO17A cells expressed high levels of thrombospondin 1 basally and addition of serum did not increase its levels (Fig. 7B), as it did in cells from the three other patients. Microarray expression analysis was performed to determine the molecular subtype as described in Materials and Methods. PriGO7A, 8 A and 9A cells were predominantly of the classical molecular subtype, while PriGO17A cells had a substantial component of mesenchymal subtype in its expression profile (Fig. 8A). Analysis of the TCGA database using cBioportal^38, 39^ showed that levels of thrombospondin mRNA levels are significantly higher in the mesenchymal molecular subtype compared to all other subtypes (Fig. 8B). NF1, which functions as a negative regulator of the Ras/Raf/Mek/Erk signalling pathway, is frequently inactivated by mutation in the mesenchymal subtype^1^. In TCGA data, levels of THBS1 mRNA positively correlated with both phosphorylated Raf1 (S338) (p = 0.0103) and phosphorylated MEK1 (S217) (p = 1.4 × 10^−3^). To determine whether there was a causative relationship between Ras pathway activation and THBS1 levels, PriGO17A cells were treated with the MEK inhibitor U0126 and secreted THBS1 levels were measured. U0126 significantly reduced the expression of thrombospondin 1 in PriGO17A cells (Fig. 8C). This indicates that Ras pathway signaling is driving the high levels of thrombospondin 1 in these cells. Pretreatment of PriGO17A cells with U0126 resulted in higher TGFβ-stimulated SMAD2 phosphorylation, indicating that Ras pathway signaling also repressed TGFβ pathway activation in these cells (Fig. 8D). PriGO17A cells pretreated with U1026 also resulted in a significant increase in the percentage of SAβgal positive cells after TGFβ treatment (Fig. 8E), indicating that Ras pathway activation also prevented induction of senescence by TGFβ in these cells. U0126 treatment did not enhance TGFβ-stimulated SMAD2 phosphorylation (Fig. 8F) or senescence induction (Fig. 8G) in PriGO8A cells.

## Discussion

Serum has been frequently used to demonstrate the multilineage differentiation potential of primary glioblastoma cells. We show here that this differentiation can be aberrant, in that many cells are positive for markers of both astrocytic and neuronal lineages. This is in agreement with previous findings^22^. We show here that these cells that have undergone “aberrant differentiation” show multiple markers of senescence. Senescence has been defined as a state of “irreversible, stress-induced growth arrest”^9^. A challenge in the field is that no one marker, or even one set of markers, appears to conclusively identify a cell as senescent. Here, decreased growth and EdU incorporation, increased SAβgal activity, morphological changes, increased p21 and an increase in PML bodies supported the conclusion that these cells were senescent. Treatment of PriGO8A cells with BMP4 induced them to undergo differentiation along the astrocytic lineage that was not aberrant in so far as there was no co-expression of neuronal markers. BMP4 treatment did not cause a rise in SAβgal activity, showing that this is not a feature of differentiation *per se*. We have shown previously that PriGO8A cells can also be induced to differentiate preferentially along the neuronal lineage by expression of PTEN or a constitutively active version of the tumour-suppressor protein Lgl1^23^. Thus, although PriGO8A cells are capable of selectively undergoing differentiation along astrocytic or neuronal lineages, serum induces an alternate state that more closely resembles senescence. Therefore some caution should be used in the use of serum as a reagent to induce “differentiation” of primary glioblastoma cells.

We did not detect an active DNA damage response in PriGO8A cells that had undergone serum-induced senescence, as assessed by immunofluorescence for γH2AX foci. The DNA damage response has been proposed to be an essential mediator of both replicative and oncogene-induced senescence^40, 41^. However there are multiple reports of DNA damage response-independent (or, more precisely, DNA double strand break-independent) senescence^17, 18, 42–45^. Recent evidence suggests that the DNA double strand break initiation of senescence may be restricted to fibroblasts, while epithelial cells undergo senescence in response to sustained DNA single strand breaks^46^. This mechanism involves downregulation of PARP1 at the mRNA level; this is not evident in our microarray expression analysis, suggesting that this is not the mechanism by which primary glioblastoma cells undergo senescence. PriGO8A cells did not undergo senescence in response to radiation. This likely reflects an impaired p53/p16-dependent senescence response to DNA damage. This resistance to radiation-induced senescence makes the finding of senescence induction by serum more intriguing. In the absence of fully functional p53/p16-dependent pathways, senescence induction may be mediated in part by non-p53-dependent mechanisms of p21 induction^47^.

The finding that PriGO8A primary glioblastoma cells underwent senescence in response to serum exposure is similar to the findings of a previous study that showed that mouse embryonic stem cells underwent senescence in response to serum^48^. In this paper, mouse embryo cells exposed to serum underwent a growth crisis, which was followed by the appearance of established aneuploid cells. This is similar to the response to serum of primary glioblastoma cells described by Lee *et al*., where in response to serum exposure these cells underwent a growth delay followed by the appearance of cells with altered chromosome numbers^22^. Many of the permanent glioblastoma cell lines currently in use may be cells that have escaped senescence imposed by serum exposure. Demidenko and Blagosklonny have also described induction of senescence by serum in human fibrosarcoma cells that are cell cycle arrested by artificial expression of p21^49^. They suggest that signals that enhance cell growth (*i.e*. an increase in cell mass) in a cell cycle-arrested context promote senescence.

Microarray expression analysis suggested that the response to serum was due to activation of the TGFβ pathway in PriGO8A cells. This was supported independently by both gene ontology analyses and transcription factor data. The finding that serum exposure induced the activation of SMAD2, a key downstream mediator in the TGFβ pathway, confirmed this, as did the finding that TGFβ was able to induce senescence in PriGO8A cells. In addition, TGFβ-induced senescence has been reported for other cell types^50^. TGFβ has a complex role in cancer pathogenesis, exhibiting both cancer repressive and cancer promoting activities^34^. Cancer repressive activities include the ability of TGFβ to induce cytostasis or apoptosis in some cells. Cancer promoting activities include the ability of TGFβ to promote invasion by inducing an epithelial-to-mesenchymal transition and the ability of TGFβ to induce an immunosuppressive state in tissue-resident macrophages. An important consideration in TGFβ pathway signalling is that differences in the strength and duration of signals can result in completely different cellular responses^51, 52^. In these experiments, it is a robust, acute activation of the TGFβ pathway that leads to senescence. A mechanism for TGFβ receptor stabilization in glioblastoma has been described^53^. This could potentially enhance the effects of acute TGFβ pathway activation in these cells.

The activation of the TGFβ pathway in PriGO8A cells was dependent on the basal expression of thrombospondin 1. Thrombospondin 1 is well-known as an activator of latent TGFβ, the form of TGFβ that is present in serum. The requirement for thrombospondin 1 was supported by the findings that a peptide inhibitor of thrombospondin 1 could block TGFβ pathway activation by serum, as could siRNA to thrombospondin 1. In addition, analysis of two additional patient glioblastoma cultures (PriGO7A and 9A) showed that their susceptibility to senescence induction by serum roughly corresponded to their basal levels of thrombospondin 1 expression. In PriGO7A cells, which express barely detectable levels of thrombospondin 1 basally, serum did not induce senescence. The requirement for thrombospondin 1 in serum-induced senescence could be bypassed by treating these cells directly with active TGFβ. Although PriGO7A, PriGO8A and PriGO9A senescence induction by serum corresponded to basal levels of thrombospondin expression, this was not the case in PriGO17A cells. PriGO17A cells showed very high basal expression of thrombospondin 1, but were completely resistant to senescence induction by serum. Microarray expression analysis of PriGO17A cells showed that they had a substantial mesenchymal type gene expression signature, which was absent in the cultures from other patients, which were predominantly classical. High thrombospondin 1 expression appears to be a general feature of the mesenchymal subtype in glioblastoma, based on analysis of the TCGA database. The mesenchymal subtype often has inactivating mutations in NF1, a negative regulator of Ras pathway signaling. The correlation of high thrombospondin levels with phosphorylated Raf1 and MEK1 in the TCGA database, along with the effects of U0126 on thrombospondin levels in PriGO17A cells, show that the high levels of thrombospondin are being driven by Ras pathway activation in these cells, rather than by TGFβ signalling. Ras pathway signaling also repressed activation of SMAD2 in these cells. Shifts in the migration of phosphorylated and total SMAD2 on SDS-PAGE suggest that this may involve direct modification of SMAD2. Inhibition of SMAD2 function by ERK phosphorylation has been described previously^54, 55^. Inhibition of Ras pathway signaling restored the ability of these cells to undergo TGFβ-induced senescence, presumably because of the restoration of strong SMAD2 activation.

Cancer cells develop mechanisms to overcome senescence in order to perpetuate their malignant phenotype. In glioblastoma, over 80% of cases have mutations in the TERT promoter that enhance telomerase expression^15, 56, 57^, which confers resistance to replicative senescence. About 60% of glioblastomas have inactivation of CDKN2A by homozygous deletion^57^ and the microarray expression analyses done here are consistent with PriGO7A and PriGO8A cells having CDKN2A deletions. This generally compromises both Rb and p53 function and would therefore be predicted to confer resistance to oncogene-induced senescence. This study shows that, in spite of these resistance mechanisms, primary glioblastoma cells retain the capacity to undergo senescence in response to acute activation of the TGFβ pathway. This pathway was common to glioblastoma cells isolated from four different patients. However the mechanisms of upstream signaling varied significantly between patients. Further study of the mechanism of this senescence induction pathway and comparison with normal brain cell behaviour may identify common glioblastoma cell-selective mediators of senescence induction that could serve as therapeutic targets.

## Materials and Methods

### Antibodies and reagents

TUJ1 rabbit monoclonal was from Covance (Princeton, NJ, USA product number MRB-435P). GFAP mouse monoclonal was from Sigma-Aldrich (Oakville ON Canada, product number G3893). Nestin mouse monoclonal antibody (product number MAB1259), recombinant BMP4 and recombinant TGFβ were from R&D Systems (Minneapolis MN, USA). p21 mouse monoclonal antibody, pSMAD2 rabbit monoclonal antibody and SMAD2 rabbit monoclonal antibody were from Cell Signaling Technology (Danvers MA, USA, product numbers DCS60, 138D4 and D43B4). PML mouse monoclonal antibody was from Santa Cruz Biotechnology (Santa Cruz CA, USA, product number PG-M3). LSKL peptide was from AnaSpec (Fremont CA, USA). U0126 was from Tocris Bioscience (Bristol UK).

### Cell culture

Patient samples for isolation of primary glioblastoma cells were obtained after informed consent following a protocol that was approved by the Ottawa Health Science Network Research Ethics Board. Primary glioblastoma cells were isolated as described previously^23^ following the method described by Pollard *et al*.^58^. In this method, cells are grown as adherent cells on laminin-coated plates in neural stem cell media. Routine cell culture was performed in 5% O_2_, the physiologic concentration of O_2_ in the brain^59^.

### Immunofluorescence, SAβgal assays and EdU labeling

Immunofluorescence and senescence-associated β galactosidase assays were performed as described previously^17, 18, 23^. EdU labeling was done using a Click-iT EdU Alexa-Fluor 488 Labeling Kit from ThermoFisher Scientific.

### Radiation treatments

Cells were irradiated in a Pantak cabinet X-Ray unit, operating at 250 kVp, with a dose rate of approximately 80 cGy per minute.

### Microarray expression analyses and bioinformatics

Total RNA was isolated from cells using Qiagen RNeasy Plus mini kits. Expression analysis was done using Affymetrix Human Gene 2.0 ST arrays at StemCore Laboratories (Ottawa, Canada) with one sample per condition. Full microarray data will be deposited in the Gene Expression Omnibus (GEO) database (http://www.ncbi.nih.gov/geo/). To assess the likely glioblastoma subtypes of our samples, we downloaded the file TCGA_unified_CORE_ClaNC840.txt from https://tcga-data.nci.nih.gov/docs/publications/gbm_exp/. This file contains the normalized expression levels of 840 genes, 210 each for proneural, neural, classical and mesenchymal subtypes, across 173 tumor samples. Of those tumor samples, 53 are proneural, 26 are neural, 38 are classical and 56 are mesenchymal. To relate this to our own data, we first downloaded the annotation file for our microarray chip, HuGene-2_0-st-v1.na35.hg19.probeset.csv.zip, from Affymetrix’s online technical support page (http://www.affymetrix.com/support/technical/byproduct.affx ? product = hugene-1_0-st-v1_. We used that file to associate the 840 Gene IDs in the TCGA file to transcript IDs in our microarray report. This allowed us to find one or more transcript IDs for all but 73 of the 840 Gene IDs. Transcript IDs for an additional 63 Gene IDs were found by using online databases, primarily GeneCards (http://www.genecards.org/), to identify alternative or retired Gene IDs matching the TCGA Gene IDs. Those alternative IDs were then found in the Affymetrix file. The remaining 10 Gene IDs from the TCGA signature, for which no alternative symbol could be found that matched a transcript ID in the Affymetrix documentation, were ignored in the remainder of the analysis. For 51 Gene IDs, we found multiple transcript IDs purportedly reporting expression for that gene. In such cases, we arbitrarily chose the numerically lowest transcript ID to represent the gene. We correlated the expression values of every TCGA sample to every other TCGA sample, and to each of our samples. We then plotted heatmaps of these correlation coefficients. From those heatmaps, the within-subtype similarities of the TCGA samples are visually apparent, as are the similarities of our samples to the TCGA samples representing the four subtypes.

### ELISA

ELISA assays for thrombospondin 1 were performed using human thrombospondin 1 ELISA kits from RayBiotech (Norcross GA, USA) following the manufacturer’s recommendations.

### RNA interference

RNA interference to deplete cells of thrombospondin 1 was done as described previously^60^. siGenome Human THBS1 siRNAs were purchased from Dharmacon (Lafayette, CO, USA) and had the target sequences GGACUGCGUUGGUGAUGUA and GUACAGAAACGUAGUCGUC.

### Statistics

SigmaPlot12 software was used for statistical analyses. Comparisons between two groups were done using t-tests for normally distributed data with equal variance. For groups that were not normally distributed or that did not show equal variance, the Mann-Whitney Rank Sum test was used. For comparisons between multiple groups the Kruskal-Wallis One Way Analysis of Variance on Ranks and the All Pairwise Multiple Comparison Procedures (Dunn’s Method) were used. A p value < 0.05 was considered significant.

## Electronic supplementary material

Supplementary data
