# Supplementary material for: Induction of senescence in primary glioblastoma cells by serum and TGFβ
Source: Sci Rep. 2017 May 19;7:2156. doi: 10.1038/s41598-017-02380-1 (PMC5438350; doi:10.1038/s41598-017-02380-1)
Supplement: Supplementary file 1 — Supplementary data [file 41598_2017_2380_MOESM1_ESM.pdf]

Supplementary Figures for:

Induction of senescence in primary glioblastoma cells by serum and TGFβ

Ritesh Kumar, Alexander Gont, Theodore J Perkins, Jennifer E L Hanson and Ian AJ Lorimer

Supplementary Figure S1. A. Representative images of γH2AX immunofluorescence in WI38 cells with and without radiation treatment (as a positive control) and PriGO8A cells with and without serum treatment. Quantitative data are shown in Figure 3A. B. PriGO8A cells were treated with the indicated doses of radiation. Floating and adherent cells were harvested at the indicated time points. Total and live cells counts were determined using a Vi-Cell XR cell counter (Beckman Coulter, Mississauga, ON, Canada).

Supplementary Figure S2. EdU incorporation in PriGO9A and PriGO17A cells without and with serum exposure. Data in bar graphs are from five randomly selected fields per condition. * indicates $p<0.05$ by the Mann-Whitney Rank Sum test.

Figure S1.

A.

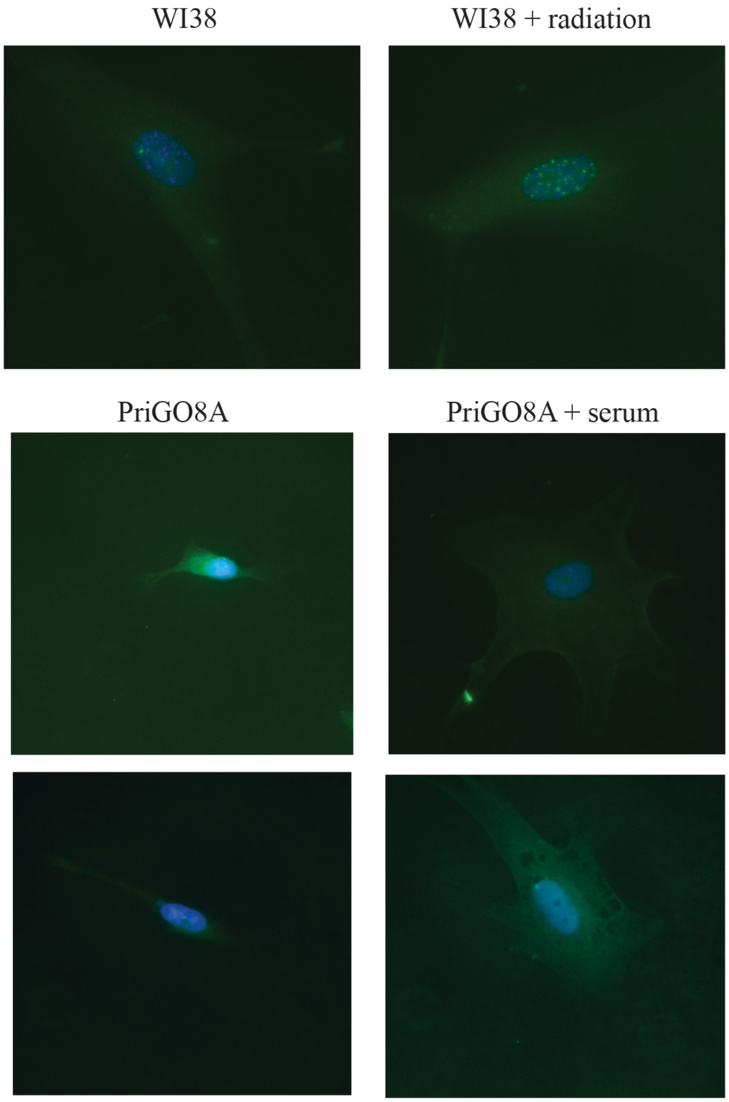
WI38
WI38 + radiation
PriGO8A
PriGO8A + serum

B.

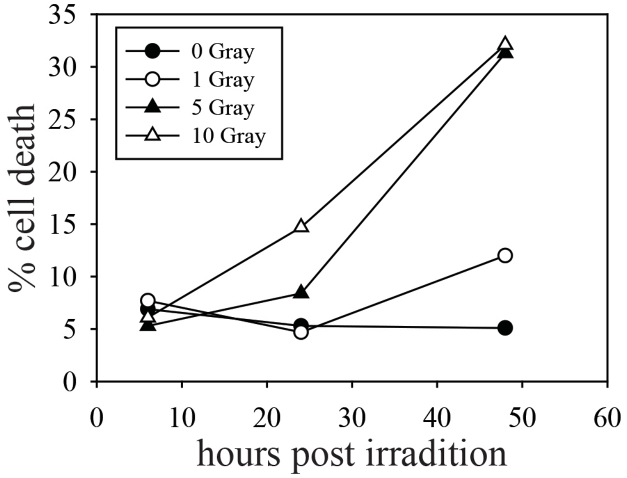
0 Gray
1 Gray
5 Gray
10 Gray
% cell death
hours post irradition
0
5
10
15
20
25
30
35
0
10
20
30
40
50
60

Figure S2.

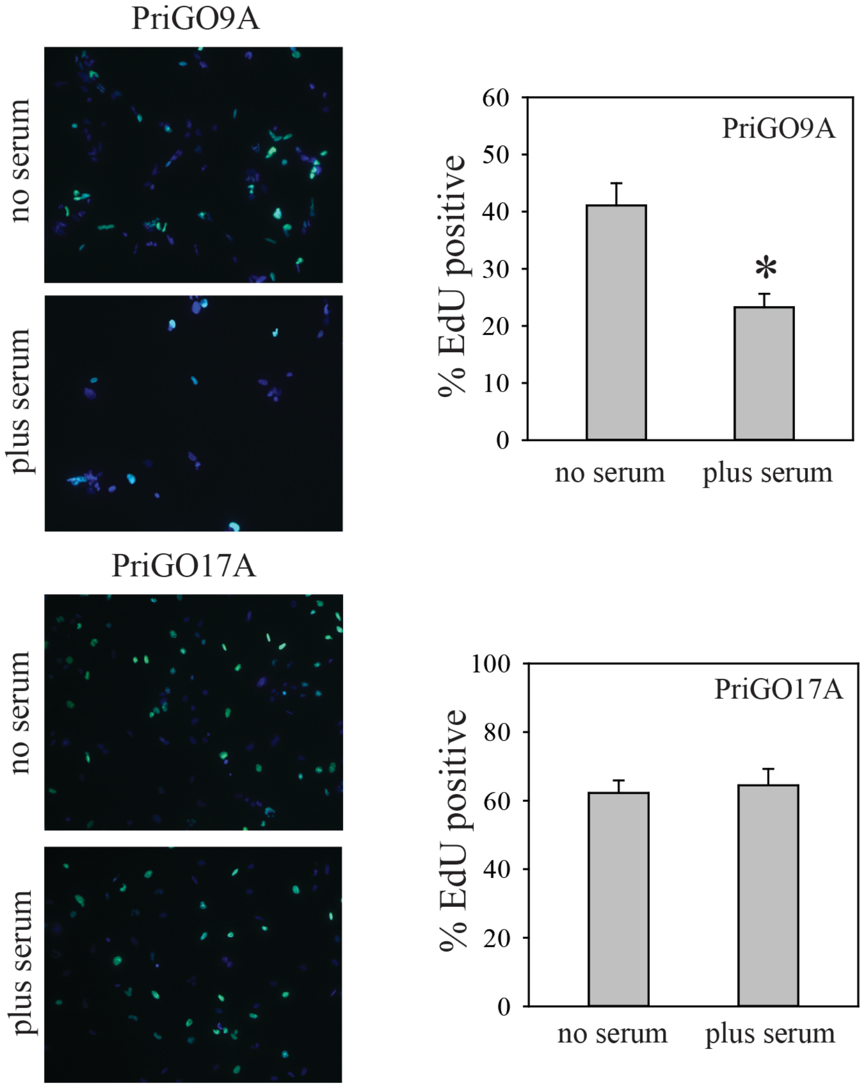
PriGO9A
no serum
plus serum
60
50
40
30
20
10
0
% EdU positive
PriGO9A
*
no serum
plus serum
PriGO17A
no serum
plus serum
100
80
60
40
20
0
% EdU positive
PriGO17A
no serum
plus serum
